# Influence of pharmacological education on perceptions, attitudes and use of dietary supplements by medical students

**DOI:** 10.1186/s12906-017-2031-6

**Published:** 2017-12-11

**Authors:** Z. Stanojević-Ristić, S. Stević, J. Rašić, D. Valjarević, M. Dejanović, A. Valjarević

**Affiliations:** 1 Department of Pharmacology and Toxicology, Faculty of Medicine, University of Kosovska Mitrovica, Anri Dinana bb, Kosovska Mitrovica, 38220 Serbia; 2 Department of Mathematics, Faculty of Sciences, University of Kosovska Mitrovica, Lole Ribara 29, Kosovska Mitrovica, 38220 Serbia; 3 Department of Physiology, Faculty of Medicine, University of Kosovska Mitrovica, Anri Dinana bb, Kosovska Mitrovica, 38220 Serbia; 4 Deparment for Management of Science and Technology Development, Ton Duc Thang University, Ho Chi Minh City, Vietnam; 5 Faculty of Environment and Labour Safety, Ton Duc Thang University, Ho Chi Minh City, Vietnam

**Keywords:** Dietary supplements, Pharmacological education, Dietary supplements usage, Perception of risk, Medical students

## Abstract

**Background:**

The ready availability and use of dietary supplements (DS) by the public means that healthcare professionals require education in this area. In the Republic of Serbia, education related to use of DS is included in undergraduate medical training and it is therefore important to assess the effectiveness of this education. The aim of our survey was to investigate the influence of pharmacological education on the use, attitudes and perceptions of risks associated with DS among medical students.

**Methods:**

Medical students at the University of Kosovska Mitrovica participated in the survey. Three hundred eighty questionnaires were distributed, yielding a response rate of 89% (*n* = 334). Data were categorized by year of study, completion of a one-year course in pharmacology and having passed the final exam. The results were compared between 192 (58%) medical students educated in pharmacology (MSEP) and 142 (42%) medical students not educated in pharmacology (MSNEP). The questionnaire was divided into 4 parts: socio-demographic and lifestyle/behavioral characteristics, use of DS, attitudes about efficacy, safety and perception of risk due to DS use. Chi-square test, Student’s t-test, and Mann-Whitney U test were used for statistical analysis.

**Results:**

About 53% of respondents used some form of DS. Attitudes regarding the safety of DS consumption showed a difference between the groups. MSEP were more likely to agree that DS have the potential to cause adverse reactions (Likert scale mean 4.1 vs. 3.5, *p* < 0.001) as well as interactions with conventional drugs (Likert scale mean 4.2 vs. 3.2, *p* < 0.001) than MSNEP. Finally, MSEP ranked St. John’s wort and ginkgo as the most dangerous DS, but creatine and vitamin C were both ranked as relatively safe. Conversely, MSNEP considered ginkgo and vitamin C the most harmful DS, claiming that omega-3 fatty acids and vitamin D had the least hazardous side effects.

**Conclusion:**

Our results showed that pharmacological education gives young medical students a better understanding of the risks of DS-drug interactions and potential adverse effects. However, their overall attitudes and perception of risk indicate the need for further education.

**Electronic supplementary material:**

The online version of this article (doi: 10.1186/s12906-017-2031-6) contains supplementary material, which is available to authorized users.

## Background

Dietary supplements (DS) are used by millions of people worldwide. They were recognized as a special group of products in the USA in 1994, and in the European Union countries (EU) in 2002. The 1994 Dietary Supplement Health and Education Act (DSHEA) [1], approved by the USA Food and Drug Administration (FDA), defined DS as a specific category of food intended to supplement the diet, but not intended to treat diseases or disorders of the human body. As a result of DSHEA, DS are not subjected to the same rigorous regulations and testing as products classified as prescription drugs.

In the EU, the EU Directive 2002/46/EC [2] was the first DS regulatory act to regulate vitamin and mineral supplements. Regulations regarding herbal DS have not yet been standardized and they are a part of different national legislations. In the Republic of Serbia, DS were regulated in 2010 by the new regulation on health safety of dietary products, [3] in line with EU regulations. Herbal medicines are classified as drugs according to the law on medicines and medical devices [4], but there are also numerous herbal DS on the market.

Since the end of the twentieth century, when the current regulations were established, the DS consumption has continued increasing. Data published from the German National Nutrition Survey II [5] showed that 16-19% of German adolescents, 14-18 years of age, reported the use of DS. The usage was more common among 18-24-year olds (21%) and increased with age. An investigation at five American universities has shown that approximately 66% of students reported having used DS once a week in the 6 months preceding the survey [6]. Research at the University of Niš (Serbia) indicated that 68% of interviewed students used DS [7]. Among the DS users, there were more medical (88%) than non-medical (65%) students.

There are many reasons for the growing popularity of DS. Irregular and inappropriate nutrition, insufficient physical activity, the pace of lifestyle, and stress are considered some of the causes of increased use of DS in order to achieve and maintain good health [7]. Changes in the regulatory status of DS have reduced or eliminated regulatory requirements that existed before DSHEA [8]. Today, DS are readily available in greater variety than previously and can easily be obtained via the internet [9].

University students may differ from the general population in DS use related to their age and socioeconomic status [10]. University life includes participating in activities such as attending class, studying, sports activities, as well as tobacco and alcohol consumption, and may encourage medical students to use DS [11, 12]. The high prevalence of iron deficiency anemia might be related to lifestyle of female students as well as to their dietary habits [13]. Saudi university students do not eat breakfast at home and the unhealthy food choices on campus may contribute to specific nutritional deficiencies [13].

DS are often taken without consultation with healthcare professionals, as they can be purchased without prescriptions at low cost. Customers may not have enough knowledge about the possible harmful effects that the DS might have. They often neglect to consult their physicians and/or pharmacists about DS consumption. Consequently, identifying the cause of specific symptoms or of their worsening is often difficult.

It is recognized that even vitamins and minerals can cause adverse reactions [14]. There is evidence that certain DS may cause adverse reactions such as liver and kidney damage, heart attack or death [15–18]. Potentially negative DS-drug interactions are also well documented [19, 20]. Drugs such as warfarin, insulin, aspirin, digoxin and ticlopidine have the most reported interactions with DS, while St. John’s wort, calcium, magnesium, iron and ginkgo have the greatest number of documented interactions with conventional drugs [20, 21]. It is well known that use of calcium and magnesium may decrease the absorption of antibiotics such as tetracyclines and fluoroquinolines [22], and St. John’s wort affects the acceleration of drug elimination [23].

Medical students in Serbia are introduced to medicinal herbs and DS in pharmacology lectures in their third year of study. During the general and specialized pharmacology courses, medical students are informed about the risks of DS use, which includes adverse reactions and DS-drug interactions.

The main aim of our survey was to investigate the influence of pharmacological education on the medical students, their DS use, attitudes towards DS, and perceptions of risk of adverse reactions.

## Methods

### Setting and sample

The survey was carried out in March 2017 at the Faculty of Medicine, University of Kosovska Mitrovica. The data were collected by a cross-sectional survey, using an anonymous questionnaire. Participation was voluntary. The questionnaire and study protocol were approved by the Ethics Committee of the Faculty of Medicine, University of Kosovska Mitrovica, Serbia.

### Participants

The respondents were medical students in the first, second, fourth, fifth and sixth year of study. Students in the third year of study who attended a course in pharmacology during the study were not included in the survey. The total number of respondents was 380, of whom 46 were excluded because of incomplete data. Stratified sampling was performed according to the year of study and pharmacological education level. Pharmacological education is defined as attending pharmacology lectures and training in the pharmacology course and having passed the final exam (see Additional file 1). The results were divided into two groups. The first group consisted of students in the first and second year of medical training who had not received pharmacology lessons, and thus had no pharmacological education. The second group consisted of students from the fourth, fifth and sixth year of study who had received pharmacology education and training.

### Questionnaire

The questionnaire was based on literature and similar surveys previously conducted [24–26]. The data about commonly available DS were gathered from local pharmacies, health food stores and regular convenience stores.

The students were invited to participate in the survey during the time between classes at the medical school. The paper-based questionnaire was designed so that the respondents could select more than one answer, or enter the appropriate data, involving minimal writing for participants. All instructions needed for completion were written on the questionnaire. One of the research team members first explained the nature of the study and then distributed consent forms and questionnaires to students. The students filled in the questionnaires independently, in the presence of research team members who were available if assistance was required.

The questionnaire included 34 items, divided into four sections.

The first section included information about the socio-demographic characteristics of students and the lifestyle/behavioral factors. These included age, gender, family income, tobacco consumption, vigorous physical activity ≥3 times per week and self-reported health.

The second section contained questions on all DS used in the previous 12 months. The list included 15 vitamins and minerals, and 20 nonvitamins and nonminerals (NVNM). Vitamins and minerals investigated included vitamins A, C, D, E, K, B complex, B6, B12, folic acid, calcium, magnesium, selenium, iron, zinc and iodine. The nonvitamin and nonmineral DS included were echinacea, *Ginkgo biloba*, ginseng, ginger, valerian, garlic, St John’s wort, aloe, creatine, protein powder, lecithin, melatonin, glucosamine/chondroitin, coenzyme Q10, probiotics, fish oil, omega-3 fatty acids, bee pollen and propolis. Respondents were asked to select reasons for DS used from 10 possible reasons, such as to improve general health, strengthen immunity, improve nutrition, etc. (see Additional file 1). Because of possible concomitant use with DS, respondents were asked about the prescription drugs and over-the-counter (OTC) medications used in the previous 12 months. In addition, respondents were asked about adverse reactions to DS. Also, the students were supposed to state whether they consider education about DS during their studies necessary.

The third section included questions related to attitude, efficacy and safety of DS used. Respondents selected from a 5 point Likert scale which ranged from strongly disagree (1), disagree (2), neutral/not sure (3), agree (4) to strongly agree (5). Survey items in this area evaluate respondents’ perceptions of the efficacy of DS in prevention and treatment of diseases, as well as their attitudes regarding the potential harmful effects and interaction with medications. Respondents were also asked about the importance of informing physicians about DS use (see Additional file 1).

The fourth section investigated the respondents’ perception of risk of adverse reactions to DS. DS evaluated included: St. John’s wort, ginkgo, vitamin C, vitamin D, iron, calcium, omega-3 fatty acids and creatine. A visual-analogue scale (VAS) was used to define a score for the perceived risk of adverse reactions. Since the VAS was a horizontal line 10 cm in length, the perceived risk of adverse reactions was considered as a quantitative score ranging from 0 to 10 (0 being the minimum and 10 being the maximum risk).

### Statistical analysis

The data analysis was conducted using the SPSS computer software package version 21.0. Descriptive statistics (mean, standard deviation, median and quartiles) were used to describe continuous variables. Frequency statistics (number and percentage) were used to describe categorical variables. The differences among the general characteristics, as well as the use of DS, were analyzed by the Chi-square test (for categorical variables). Due to the scale of measurement, the scores were compared using Student’s t-test and Mann-Whitney U test. Multivariate logistic regression analysis was conducted to determine whether there was an independent relationship between DS use and demographic/lifestyle characteristics, along with conventional drug use. Results were presented in terms of adjusted odds ratios and 95% confidence intervals. Differences were considered to be significant if the *p* value was less than 0.05.

## Results

The response rate for sample respondents was 89% (334/380) of 380 participants. Among the 334 respondents who completed the full questionnaire, 69 (21%), 73 (22%), 70 (21%), 70 (21%) and 52 (16%) were in the first, second, fourth, fifth and sixth years of study, respectively. There were 192 (58%) medical students educated in pharmacology (MSEP) in the fourth, fifth and sixth years of study, while there were 142 (42%) medical students not educated in pharmacology (MSNEP) in the first and second years of study. 56% of respondents were female and 44% were male. There was a significant age difference between the groups (24 vs. 22, *p* < 0.001). Over half of the students reported a high level of family income (52%) and assessed their health as good (50%). Approximately 22% of students were tobacco users and 22% were physically active students. There were no significant differences between groups with respect to gender, family income, tobacco use, physical activity and self-reported health. The general characteristics of the respondents are summarized in Table 1.

**Table 1:** General characteristics of the medical students included in the study

Characteristics	MSEP 192 (58%)	MSNEP 142 (42%)	Total 334 (100%)	*p*-values^a^
Gender, n (%)				0.987
Male	84 (44)	62 (44)	146 (44)	
Female	108 (56)	80 (56)	188 (56)	
Age, (mean ± SD)	24 (±2)	22 (±1)	23 (±2)	<0.001^b*^
Family income, n (%)				0.544
High	100 (52)	74 (52)	174 (52)	
Moderate	82 (43)	64 (45)	146 (44)	
Low	10 (5)	4 (3)	14 (4)	
Tobacco use, n (%)				0.768
Yes	42 (22)	33 (23)	75 (22)	
No	150 (78)	109 (77)	259 (78)	
Physical activity, n (%)				0.586
Yes	44 (23)	29 (20)	73 (22)	
No	148 (77)	113 (80)	261 (78)	
Self-reported health, n (%)				0.820
Excellent	65 (34)	47 (33)	112 (33)	
Good	93 (48)	73 (51)	166 (50)	
Poor	34 (18)	22 (16)	56 (17)	

^a^ *p* values determined using chi square for categorical variables and ^b^Student’s t-test for continuous variables

^*^ *p* < 0.001 – MSEP vs. MSNEP

Table 2 presents the results regarding DS use by medical students. One hundred seventy eight respondents (53%) reported DS use, while 36 respondents (11%) had used OTC medications and 18 respondents (5%) had used prescription drugs in the previous 12 months.

**Table 2:** Consumption and reasons for use of DS by medical students

Characteristics	MSEP n (%)	MSNEP n (%)	Total n (%)	*p*-values^a^
DS use				0.337
Yes	98 (51)	80 (56)	178 (53)	
No	94 (49)	62 (44)	156 (47)	
OTC medications use				0.094
Yes	16 (8)	20 (14)	36 (11)	
No	176 (92)	122 (86)	298 (89)	
Prescription drugs use				0.101
Yes	7 (4)	11 (8)	18 (5)	
No	185 (96)	131 (92)	316 (95)	
Reasons for DS use				
Improve general health	90 (47)	74 (52)	164 (49)	0.344
Strengthen immunity	72 (38)	57 (40)	129 (39)	0.624
Improve nutrition	62 (32)	42 (30)	104 (31)	0.596
Enhance athletic performance	56 (29)	24 (17)	80 (24)	0.009^*^
Improve concentration	34 (18)	23 (16)	57 (17)	0.717
Increase endurance	28 (15)	23 (16)	51 (15)	0.685
Relieve stress	14 (7)	9 (6)	23 (7)	0.734
Promote weight loss	14 (7)	8 (6)	22 (7)	0.546
Prevent/relieve PMS	6 (3)	1 (1)	7 (2)	0.127
Types of DS use				
Vitamins	66 (34)	60 (42)	126 (38)	0.142
Minerals	30 (16)	34 (24)	64 (19)	0.056
Herbal DS	18 (9)	13 (9)	31 (9)	0.945
Non-herbal DS	43 (22)	31 (22)	74 (22)	0.902
Multivitamins	36 (19)	27 (19)	63 (19)	0.951
Vitamins + minerals	28 (15)	22 (16)	50 (15)	0.818
Multivitamins + minerals	37 (19)	8 (6)	45 (14)	<0.001^**^
Multivitamins + multiminerals	24 (12)	65 (46)	89 (27)	<0.001^**^

^a^ *p* values determined using chi square

^*^ *p* < 0.01; ^**^ *p* < 0.001 – MSEP vs. MSNEP

From the multi regression analysis, factors associated with DS use included physical activity (OR: 0.3 (0.1-0.9); *p* = 0.040) and prescription drugs use (OR: 5.2 (1.4-19.3); *p* = 0.013). OTC medication use was not associated independently with DS use (data not shown).

The most frequent reasons for DS use selected by the respondents were to improve general health (49%), to strengthen immunity (39%), to improve nutrition (31%) and to enhance performance (24%). The MSEP were more likely to use DS to enhance athletic performance (29% vs. 17%, *p* = 0.009) than MSNEP.

The most popular DS among the MSEP were vitamins (34%) and non-herbal DS (22%). Vitamin C (32%), propolis (24%), protein powder (16%), magnesium (15%) and vitamin B (15%) were the most frequently used DS (Table 3). MSNEP usually used multivitamins + multiminerals (46%) and vitamins (42%) as presented in Table 2. They usually consumed vitamin C (33%), vitamin B (22%), fish oil (19%), calcium (18%) and iron (17%). In addition, several statistically significant differences regarding DS consumption habits were observed. MSEP used more propolis (24% vs. 11%, *p* < 0.001), protein powder (16% vs. 8%, *p* = 0.030) and echinacea (6% vs. 0%, *p* = 0.002), but less calcium (3% vs. 18%, *p* < 0.001) and fish oil (6% vs. 19%, *p* < 0.001) compared to MSNEP. The frequency of specific DS used by respondents is given in Table 3.

**Table 3:** The most commonly used DS among medical students

Dietary supplements	MSEP n (%)	MSNEP n (%)	Total n (%)	*p*-value^a^
Vitamin C	62 (32)	47 (33)	109 (33)	0.876
Propolis	47 (24)	15 (11)	62 (19)	<0.001^***^
Vitamin B complex	28 (15)	32 (22)	60 (18)	0.061
Magnesium	28 (15)	18 (13)	46 (14)	0.617
Iron	19 (10)	24 (17)	43 (13)	0.059
Protein powder	30 (16)	11 (8)	41 (12)	0.030^*^
Fish oil	12 (6)	27 (19)	39 (12)	<0.001^***^
Calcium	6 (3)	26 (18)	32 (10)	<0.001^***^
Probiotics	12 (6)	12 (8)	24 (7)	0.441
Vitamin D	10 (5)	13 (9)	23 (7)	0.159
Creatine	10 (5)	8 (6)	18 (5)	0.865
Omega-3 fatty acid	6 (3)	9 (6)	15 (4)	0.161
Echinacea	12 (6)	0 (0)	12 (4)	0.002^**^
Ginger	4 (2)	7 (5)	11 (3)	0.150
*Ginkgo biloba*	6 (3)	3 (2)	9 (3)	0.572
Vitamin B6	4 (2)	5 (4)	9 (3)	0.422
Selenium	4 (2)	2 (1)	6 (2)	0.646
Coenzyme Q10	4 (2)	3 (2)	7 (2)	0.985
Folic acid	4 (2)	2 (1)	6 (2)	0.646

^a^ *p* values determined using chi square

^*^ *p* < 0.05; ^**^ *p* < 0.01; ^***^ *p* < 0.001 – MSEP vs. MSNEP

Only 4% of respondents reported having experienced adverse reactions after taking DS (data not shown). Adverse reactions included constipation, abdominal pain, sleep problems and nausea (data not shown).

The student attitudes regarding the efficacy and safety of DS are given in Table 4.

**Table 4:** Respondents attitudes towards DS

	MSEP mean (±SD)	MSNEP mean (±SD)	*p*-value^b^
(1 = strongly disagree, 2 = disagree, 3 = neutral/not sure, 4 = agree, 5 = strongly agree)^a^			
DS can be useful for prevention of diseases	4.1 (±1.0)	3.9 (±1.1)	0.093
DS can be useful for treatment of diseases	2.9 (±1.2)	2.6 (±1.2)	0.069
When using DS it is important to follow the manufacturer’s instructions	4.1 (±1.2)	3.8 (±1.0)	0.053
DS can cause harmful effects	4.1 (±0.9)	3.5 (±1.3)	<0.001^*^
DS adverse reactions should be reported to physicians or pharmacists	4.0 (±1.0)	3.0 (±1.2)	<0.001^*^
DS can be dangerous when combined with drugs	4.2 (±0.8)	3.2 (±1.3)	<0.001^*^
It is important to inform physicians about DS use	3.0 (±1.1)	2.8 (±1.2)	0.115
Physicians should ask patients about DS use, before prescribing drugs	4.2 (±0.9)	4.3 (±1.0)	0.284

^a^Likert-type scale

^b^ *p* values determined using Student’s t-test

^*^ *p* < 0.001 – MSEP vs. MSNEP

The MSEP and MSNEP agreed that DS can be useful in the prevention of diseases (Likert scale mean 4.1 vs. 3.9, *p* = 0.093), but they were not sure whether DS can be useful for the treatment of diseases (Likert scale mean 2.9 vs. 2.6, *p* = 0.069). Most of the medical students agreed that following the manufacturer’s instructions about DS use is of great importance (Likert scale mean 4.1 vs. 3.8, *p* = 0.053). The majority of MSNEP were not sure about the potential of DS to cause adverse reactions while MSEP thought that DS have the potential to cause adverse reactions (Likert scale mean 3.5 vs. 4.1, *p* < 0.001) and interactions with conventional drugs (3.2 vs. 4.2, *p* < 0.001). Similar results were obtained with respect to reporting adverse reactions to DS to physicians or pharmacists (Likert scale mean 3.0 vs. 4.0, *p* < 0.001).

Despite the differences in attitude towards DS safety, the majority of medical students were not sure they need to report their DS use to physicians (Likert scale mean 2.8 vs. 3.0, *p* = 0.115). However, they agreed that physicians should ask patients about DS use before prescribing drugs (Likert scale mean 4.3 vs. 4.2, *p* = 0.284).

**Fig. 1:**
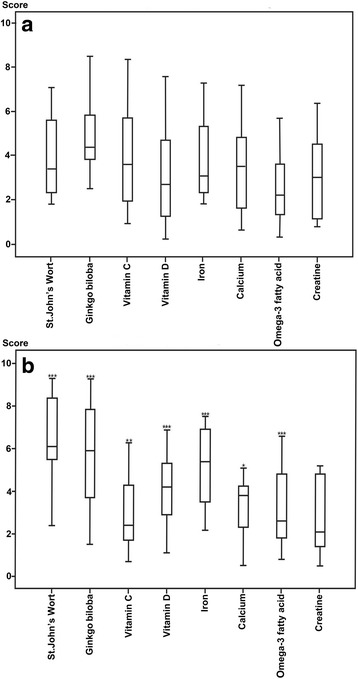
Perceived risk of adverse reactions to DS. Results are presented as median scores of perceived risk on visual analogue scales (25th – 75th centiles) by the (**a**) medical students not educated and (**b**) educated in pharmacology. *p < 0.05, **p < 0.01, and ***p < 0.001 for differences between MSNEP and MSEP

The DS were ranked by the median value (25th-75th centiles) of perceived risk of adverse reactions and presented on the visual analogue scale (Fig. 1). MSEP ranked St. John’s wort (6.1, 5.5-8.6) as the most dangerous DS, followed by ginkgo (5.9, 3.7-7.8) and iron (5.4, 3.5-6.9). Creatine (2.1, 1.4-4.8) and vitamin C (2.4, 1.7-4.3) were both ranked as relatively safe. Ginkgo (4.4, 3.5-5.8) was ranked as the most dangerous DS by MSNEP, followed by vitamin C (3.6, 1.9-5.7) and calcium (3.5, 1.6-4.9). Omega-3 fatty acids (2.2, 1.3-3.6) were in the last position.

When all DS were considered together, the mean (±SD) of the median scores of perceived risk was 4.2 ± 2.0 for MSEP and 3.7 ± 1.9 for MSNEP, which was a statistically very significant difference (*p* < 0.001).

Finally, in response to considering the need to learn about DS during their studies a necessity, 83% of the MSEP and 84% of the MSNEP answered affirmatively (data not shown).

## Discussion

This study investigated the influence of pharmacological education on the use and attitudes of DS among medical students and differences between MSEP and MSNEP in the perceived risk of adverse reactions.

According to the results of our survey, the use of DS among medical students is widespread - over the half of respondents reported having taken DS in the previous 12 months. The most common DS used were vitamins, multivitamins + multiminerals and non-herbal DS. The most frequently used specific DS included vitamin C, propolis, vitamin B, magnesium, iron, protein powder and fish oil. Compared to the MSNEP, MSEP used significantly more propolis, protein powder and echinacea, but significantly less calcium and fish oil. It has been reported that propolis enhances immune system activities [27], oxygen radical scavenging [28], antimicrobial [29] and antitumor activities [30] which may account for the popularity of these DS among MSEP.

Previous surveys also reported higher rates of DS use by students [31, 32]. Vitamin C, multivitamins and multivitamins + iron/other minerals were the most popular DS among Australian university students [31]. Echinacea, ginseng and St. John’s wort were the most frequently used nonvitamin and nonmineral DS [33, 34].

The increased use of vitamins and multivitamin + multimineral DS by students is expected because of their easier accessibility compared to herbal or other DS. In addition, Bailey et al. reported that herbal DS use is more common in older than in younger age groups [35].

Our respondents used DS to improve general health, to strengthen immunity, to improve nutrition and to enhance performance [see 6, 36]. Based on previous studies [36, 37] general reasons of health maintenance are often reported to justify the use of DS. Barnes et al. suggested that many DS users perceive supplements to be healthy, but they were not certain how the supplements influenced the functioning of their bodies [38].

In our survey, physically active respondents were more likely to use DS. Numerous studies have found а strong link between physical activity and DS use among students and adults [39, 40]. According to Gardiner et al. (2007) the use of sports supplements is concerning due to their questionable safety and efficacy [39].

Both MSEP and MSNEP believed that DS can be useful for the prevention of diseases, but were not sure whether DS can be helpful in the treatment of diseases. By definition, under the law, DS are not intended to treat or prevent any disease, although many people use DS for these purposes [41]. These findings may reflect medical students perception that DS “improve general health and strengthen immunity” and hence prevent disease. Similar results were reported by Owens et al. [25]. Furthermore, Nichter et al. (2006) reported that 2/3 of respondents would continue to use a specific DS even if it was scientifically proven to be ineffective [42].

The majority of the medical students believed that it is important to follow the manufacturer’s instructions in DS use, which is a positive trend, given that excessive doses of DS can lead to a number of disorders. For example, excessive dietary intake of vitamin A is associated with decreased bone mineral density and an increased risk of hip fracture [43].

MSEP compared to MSNEP ones expressed a higher level of concern regarding potential adverse reactions and DS-drug interactions. Similar results were obtained when they were asked about the importance of reporting adverse reactions to DS to healthcare professionals. This indicates that pharmacological education significantly affects the students’ attitudes concerning the risks of using DS and the importance of reporting adverse effects.

Approximately 4% of respondents reported having experienced adverse reactions such as nausea, constipation, abdominal pain and sleep problems [see also 9, 32]. The reasons for a relatively low occurrence of adverse reactions in these respondents could be the doses of DS used which were not measured nor duration of supplementation. In our sample, the medical students who used prescription drugs were more likely to use DS. This is a serious concern because the use of DS with conventional drugs increases the risk of adverse reactions and DS-drug interactions.

Interestingly, medical students were not sure whether they needed to inform their physicians about DS consumption, but they agreed that physicians should request such information. This corroborates a recently published cross-sectional survey by Tangkiatkumjai et al. (2013) where the respondents said that the main reason for not reporting DS use was that they were not asked about it [44].

In this paper, we investigated whether the perceived risk of adverse reactions to DS differs between the groups of medical students. The MSEP consider St. John’s wort, ginkgo and iron potent supplements could cause serious adverse effects, while creatine and vitamin C are considered safer. In contrast, the MSNEP thought that ginkgo and vitamin C were the most dangerous, and that omega-3 fatty acids and vitamin D were the least dangerous DS. Apart from the influence of pharmacological education, the difference in the perception of risk between the groups could be explained by the influence of media, too. Information on DS from a variety of media sources including television, radio, print and the internet is widely available, but the information is often contradictory and confusing [6]. Our findings showed that the most popular sources of information about supplements among the MSEP were the books/professional journals and pharmacists, for the MSNEP these were the media and friends (data not shown).

The MSEP consider creatine consumption relatively safe, although its use is associated with a risk of gastrointestinal distress, water retention and nephritis [45, 46].

Both groups of students ranked ginkgo as one of the most dangerous DS. Ginkgo, a very popular supplement taken to enhance memory, can provoke spontaneous bleeding, and warnings about this effect have been published by health authorities and the media in the last 15 years [47].

An interesting result of this survey is that MSEP showed a higher global perception of risk than MSNEP (global mean score 4.2 vs. 3.7, *p* < 0.001). If we compare these results to the study of Durrieu et al. [26] the MSEP assigned lower risk values to DS (4.2) than to drugs (5.8). This opinion is in line with the common attitude that DS are natural, relatively safe and can be used with very little caution.

Our survey was carried out on a relatively small sample and might not reflect the attitudes of all Serbian medical students. Therefore, this research should be extended to other faculties of medicine in Serbia in order to obtain better insight into the medical students’ knowledge of the benefits and risks of DS use. Also, not all of the DS available on the market were included in the survey. Future studies could provide more detailed information about medical students’ knowledge and perception of DS-related risk of adverse reactions and DS-drug interactions.

## Conclusion

The growing interest in DS, from within the pharmaceutical industry and among consumers means that there is a need to involve all stakeholders in the society to ensure a safer and more rational utilization of these products. Healthcare professionals, nutritionists and sports workers have a special role in this process. Informing health care physicians about DS is a necessary and integral part of their education.

Pharmacy students have many more lectures on DS compared to medical students. It is extremely important to professionally teach not only pharmacy students, but also medical students about both the benefits and the risks of DS use.

It is necessary for medical students (during their pharmacology education and, of course, during studies in general), to understand the complexity of their future work as healthcare professionals. They have to be aware of the importance of directly asking the patients about DS use before prescribing conventional drugs, in order to reduce the risk of potential harmful interactions in practice. Their knowledge about the consequences, opportunities and restrictions regarding the use of DS is essential for effective supplementation.

This research shows that the current level of information related to using DS among medical students should be substantially increased. With medical students themselves being significant consumers of DS, their attitudes and perceptions of risk indicate the need for expert education, and in the future this issue might be considered with much more attention. Because of that, the Faculty of Medicine should improve its programs and curricula to include more expert statements regarding DS.

## Additional file

Additional file 1: Dietary supplements questionnaire. The questionnaire used to examine participant perceptions and attitudes about DS as well as the use of DS. (DOCX 65 kb)

## Abbreviations

DS: Dietary supplements
DSHEA: Dietary Supplement Health and Education Act
EU: European Union
FDA: USA Food and Drug Administration
MSEP: Medical students educated in pharmacology
MSNEP: Medical students not educated in pharmacology
NVNM: Nonvitamins and nonminerals
OR: Adjusted Odds Ratio in Multivariate Logistic Regression Analysis
OTC: Over-The-Counter
SPSS: Statistical Package for Social Sciences
USA: United States of America
VAS: Visual-analogue scale
