# Supplementary material for: Influence of pharmacological education on perceptions, attitudes and use of dietary supplements by medical students
Source: BMC Complement Altern Med. 2017 Dec 11;17:527. doi: 10.1186/s12906-017-2031-6 (PMC5725837; doi:10.1186/s12906-017-2031-6)
Supplement: Additional file 1: — Dietary supplements questionnaire. The questionnaire used to examine participant perceptions and attitudes about DS as well as the use of DS. (DOCX 65 kb) [file 12906_2017_2031_MOESM1_ESM.docx]

**Additional file 1**

**DIETARY SUPPLEMENTS QUESTIONNAIRE**

Please read each questions, insert X in the appropriate field or scale, circle one or more answers or enter the appropriate data. Please, answer all questions.

**I Demographic and lifestyle/behavioral characteristics**

1. Gender M F

2. Year of birth ______________

3. Family income High Moderate Low

0

4. Tobacco use Yes No

0

5. Vigorous physical activity

≥3 times per week Yes No

0

6. Health status Excellent Good Poor

0

7. Year of study ______________

8. Passed the course in

pharmacological training Yes No

0

9. Passed the exam in

pharmacology Yes No

0

**II Dietary supplements (DS) and drugs used in the past 12 months**

10. DS used Yes No

0

11. OTC drugs used Yes No

0

12. Prescription drugs used Yes No

0

13. Reasons for DS used (select one or more answers)

1. Improve general health
2. Strengthen immunity
3. Improve nutrition
4. Enhance athletic performance
5. Improve concentration
6. Increase endurance
7. Relieve stress
8. Promote weight loss
9. Prevent/relieve PMS
10. Other reasons ___________________

14. Types of DS used in the past 12 months (select one or more answers)

1. Vitamins
2. Minerals
3. Herbal DS
4. Non-herbal DS
5. Vitamins + minerals
6. Multivitamins
7. Multivitamins+minerals
8. Multivitamins+multiminerals
9. Vitamins+minerals+herbal DS
10. Multivitamins+multiminerals+herbal DS
11. Vitamins+minerals+non-herbal DS
12. Multivitamins+multiminerals+non-herbal DS

15. Specific DS used in the past 12 months (select one or more answers)

| *Vitamins* | *Minerals* | *Herbal DS* | *Non-herbal DS* |
| --- | --- | --- | --- |
| - 1. Vitamin A   2. Vitamin C   3. Vitamin D   4. Vitamin E   5. Vitamin K   6. Vitamin B   7. Vitamin B6   8. Vitamin B12   9. Folic acid | - 1. Calcium   2. Magnesium   3. Selenium   4. Iron   5. Zinc   6. Iodine   7. __________ | - 1. Echinacea   2. Ginkgo biloba   3. Ginseng   4. Ginger   5. Valerian   6. Garlic   7. St John's wort   8. Aloe   9. ___________ | 1. Creatine 2. Protein powder 3. Lecithin 4. Melatonin 5. Glucosamine/chondroitin 6. Coenzyme Q10 7. Probiotics 8. Fish oil 9. Omega-3 fatty acids 10. Bee pollen 11. Propolis 12. ______________ |

16. Did DS caused a adverse reactions? Yes No

0

0

17. If Yes, please specify? _____________________________

18. Do you think that for medical students is

necessary training about the DS,

during the study? Yes No

0

0

**III Attitudes about the efficacy and safety of DS use (**insert X into the appropriate field**)**

|  | *Strongly disagree* | *Disagree* | *Neutral/*  *Not sure* | *Agree* | *Strongly agree* |
| --- | --- | --- | --- | --- | --- |
| 19. DS can be useful for prevention of disease |  |  |  |  |  |
| 20. DS can be useful for treatment of diseases |  |  |  |  |  |
| 21. When using DS it is important to follow the manufacturer's instructions |  |  |  |  |  |
| 22. DS can cause harmful effects |  |  |  |  |  |
| 23. DS adverse reactions should be reported to physicians or pharmacists |  |  |  |  |  |
| 24. DS can be dangerous when combined with drugs |  |  |  |  |  |
| 25. It is important to inform physicians about DS use |  |  |  |  |  |
| 26. Physicians should ask patients about DS use, before prescribing drugs |  |  |  |  |  |

**IV Perception of risk for DS adverse reactions (**mark X on the scale**)**

Minimal risk Maximal risk

27. St. John's wort ______________________________________________

0 10

Minimal risk Maximal risk

28. Ginkgo biloba ______________________________________________

0 10

Minimal risk Maximal risk

29. Vitamin C ______________________________________________

0 10

Minimal risk Maximal risk

30. Vitamin D ______________________________________________

0 10

Minimal risk Maximal risk

31. Iron ______________________________________________

0 10

Minimal risk Maximal risk

32. Calcium ______________________________________________

0 10

Minimal risk Maximal risk

33. Omega-3 fatty acids ______________________________________________

0 10

Minimal risk Maximal risk

34. Creatine ______________________________________________

0 10
